# Racial Disparities among Asian American, Native Hawaiian, and Other Pacific Islander Patients with Cancer Who Refuse Recommended Radiation Therapy or Surgery

**DOI:** 10.3390/cancers15133358

**Published:** 2023-06-26

**Authors:** Brianna Lau, Paul Tominez, Jaimie Z. Shing, Jacqueline B. Vo, Erqi Pollom, Kekoa Taparra

**Affiliations:** 1 Department of Radiation Oncology, Stanford Medicine, Palo Alto, CA 94304, USA; brilau8@stanford.edu (B.L.); erqiliu@stanford.edu (E.P.); 2 School of Medicine, University of California, San Francisco, CA 94143, USA; paul.tominez@ucsf.edu; 3 Division of Cancer Epidemiology & Genetics, National Cancer Institute, National Institutes of Health, Bethesda, MD 20892, USA; jaimie.shing@nih.gov (J.Z.S.); jacqueline.vo@nih.gov (J.B.V.); 4 Affiliated Physician, Palo Alto Veterans Affairs Hospital, Palo Alto, CA 94304, USA

**Keywords:** cancer disparities, Asian Americans, Native Hawaiian and Other Pacific Islanders, surgery refusal, radiotherapy refusal

## Abstract

**Simple Summary:**

Despite the advances in radiation therapy and surgical techniques that offer significant curative potential for patients with cancer, previous studies have shown a higher treatment refusal rate among Asian Americans (AA), Native Hawaiian, and other Pacific Islander (NHPI) populations. Treatment refusal has been correlated with poorer survival outcomes. In this study, we aimed to (1) estimate and compare treatment refusal rates by disaggregated AA ethnogeographic regions (based on geographic proximity of ethnicity) and NHPI individuals, (2) assess the impact of treatment refusal on overall mortality among disaggregated AA and NHPI populations in the United States adjusted for confounders, and (3) identify predictors for treatment refusal. Results from this study unmask the racial and ethnic disparities regarding cancer treatment within AA and NHPI populations and highlight the need for data disaggregation throughout the research community.

**Abstract:**

Despite radiation therapy (RT) and surgery being the curative treatments, prior work demonstrated that the aggregated Asian American (AA) and Native Hawaiian and Other Pacific Islanders (NHPI) population refuse RT and surgery at a higher rates than other races. Given that AA and NHPI are distinct groups, data disaggregation is necessary to understand racial and ethnic disparities for treatment refusal. We aimed to (1) compare RT and surgery refusal rates between AA and NHPI populations, (2) assess RT and surgery refusal on overall mortality, and (3) determine predictors of refusing RT and surgery using the United States (U.S.) National Cancer Database. Adjusted odds ratios (aOR) and 95% confidence intervals (95%CI) for treatment refusal were calculated using logistic regression. Adjusted hazard ratios (aHR) were calculated for overall survival using Cox proportional hazard models among propensity score-matched groups. The overall rate of RT refusal was 4.8% and surgery refusal was 0.8%. Compared to East AA patients, NHPI patients had the highest risk of both RT refusal (aOR = 1.38, 95%CI = 1.21–1.61) and surgery refusal (aOR = 1.28, 95%CI = 1.00–1.61). RT refusal significantly predicted higher mortality (aHR = 1.17, 95%CI = 1.08–1.27), whereas surgery refusal did not. Predictors of RT and surgery refusal were older patient age, high comorbidity index, and cancer diagnosis between 2011–2017. The results show heterogenous treatment refusal patterns among AA and NHPI populations, suggesting areas for targeted intervention.

## 1. Introduction

### 1.1. Treatment Refusal for Cancer

Surgery and radiation therapy (RT) often represent the only curative options for patients with nonmetastatic solid tumors. However, even when recommended by oncology healthcare providers, some patients continue to refuse these proven cancer treatments. Previous United States (U.S.) data showed approximately 1–5% refusal rate for RT [1,2] and surgery [3]. Refusal of RT treatment was associated with increased cancer-specific mortality with an unadjusted decrease in median overall survival of about 75 months [2,4]. They also showed that the rates of refusal did not change over several decades regardless of radiotherapy technology improvements and toxicity reductions, pointing to the complexity of treatment refusal, such as socioeconomic and cultural attributes. As for surgery refusal, a recent cohort study on endometrial cancer found that among patients who refused surgery, their 5-year overall survival was significantly decreased (29.2% vs. 71.9%, *p* < 0.01) [5]. This finding was most notable for patients older than 40 years of age. Other studies have found that advanced age, single marital status, and Asian American (AA) race were associated with refusal of RT and surgery [4]. For this reason, it is important to understand the predictive factors of treatment refusal among patients with cancer so that culturally competent and targeted educational interventions and resources may be directed appropriately to these patients and their healthcare providers.

### 1.2. AA and NHPI Data Disaggregation

In the U.S., the only two races for which cancer is the leading cause of death are Asian American (AA) and Native Hawaiian and Other Pacific Islander (NHPI) populations [6,7,8]. Prior work has underscored that the AA and NHPI populations refuse RT and surgery at a higher rate than other races when aggregated, even after comorbid conditions and socioeconomic factors are accounted for [3,4,9]. Despite these populations historically grouped under the umbrella term “AAPI”, AA and NHPI are not a single monolithic group, and it is important to recognize the heterogeneity, health disparities, and inequities that arise due to aggregation of NHPI with AA data [10]. To date, there is limited data on RT and surgery refusal patterns with disaggregated AA and NHPI population data. The aims of this investigation were to (1) estimate and compare treatment refusal rates by disaggregated AA ethnogeographic regions and NHPI individuals, (2) assess the impact of treatment refusal on overall survival among disaggregated AA and NHPI populations in the U.S., and (3) identify predictors for treatment refusal among populations at highest risk.

## 2. Materials and Methods

### 2.1. Study Design and Patient Demographics

This U.S. hospital-based retrospective cohort study was conducted using the National Cancer Database (NCDB) [11]. Informed consent and review by the Stanford University Institutional Review Board were deemed exempt because data were de-identified and publicly available. Patients who were ≥18 years old, of AA or NHPI race, and with a confirmed diagnosis of one of the ten most common cancers in the U.S. between 2004 and 2017 were eligible for this study. Patients who had complete data for overall survival, follow-up, biopsy confirmed disease, staging, race, and RT/surgery recommended by the treating physician were included. The top 10 most common cancers in the U.S. were defined as lung, breast, colorectal, endometrial, kidney/bladder, melanoma, oral cavity, pancreatic, prostate, and thyroid cancers [12].

AA patients were disaggregated as East AA (Chinese, Japanese, and Korean), South AA (Indian and Pakistani), and Southeast AA (Cambodian, Filipino, Hmong, Laotian, Kampuchean, Thai, and Vietnamese). The NHPI population included Native Hawaiian, Micronesian, Chamorro, Guamanian, Polynesian, Tahitian, Samoan, Tongan, Melanesian, Fiji Islander, New Guinean, and other Pacific Islander patients. East AA patients were defined a priori as the referent group due to this group representing most of the AA population in this study. For RT refusal specifically, we performed a subset analysis on East AA patients further disaggregated by Chinese, Japanese, and Korean to assess whether RT refusal was more likely in specific East AA populations.

### 2.2. Variables

**Figure 1:**
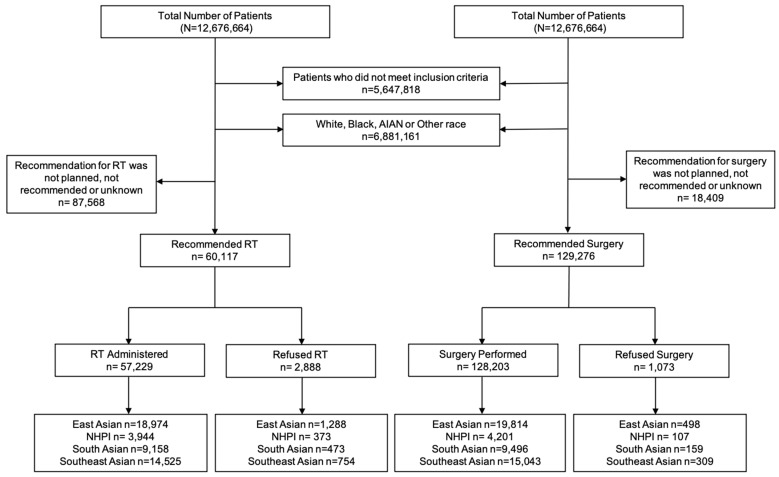
Study flowchart of patients included in this study. Patients were included for complete survival, complete follow-up, complete staging data, biopsy confirmed diagnosis, age ≥18 years, and recommended for RT or surgery as an initial treatment. Patient race included AA and NHPI. AA was stratified by East AA, South AA, and Southeast AA. The analyses were divided into patients who were recommended for RT and those who were recommended for surgery. Abbreviations: AA = Asian American; NHPI = Native Hawaiian and other Pacific Islander; RT = Radiation Therapy; AIAN = American Indian/Alaska Native.

The primary endpoint was RT and surgery refusal rate. In the NCDB, RT refusal was specifically coded as “Radiation therapy was not administered; it was recommended by the patient’s physician, but this treatment was refused by the patient, the patient’s family member, or the patient’s guardian. The refusal was noted in the patient record”. Surgery refusal was coded as “Surgery of the primary site was not performed; it was recommended by the patient’s physician, but this treatment was refused by the patient, the patient’s family member, or the patient’s guardian. The refusal was noted in the patient record” [11]. All patients included in this study were recommended for RT or surgery by their treating physician. We performed two separate analyses. One with patients who were recommended for RT and another with patients who were recommended for surgery (Figure 1). These groups were not mutually exclusive (i.e., patients who were recommended RT could have also been recommended for surgery).

The 14 covariates included in this study were race, age, sex, cancer stage (early vs. late), cancer type, income (above vs. below median), rurality (urban/rural vs. metropolitan), education (above vs. below median), insurance status, distance from the hospital (miles), comorbidities by the Charlson–Deyo Comorbidity Index, facility type, facility U.S. region, and year of diagnosis (2004–2010 vs. 2011–2017).

Early cancer stages were defined as overall Stages 0, I, or II, while late cancer stages were defined as Stages III or IV. Patient income and education were defined by the median household income and the percentage of non–high school graduates of the patient’s areas of residence from 2012 American Community Survey data, respectively [13]. The categorical years of diagnosis periods were divided by the median (2004–2010 vs. 2011–2017).

### 2.3. Statistical Analysis

Unadjusted descriptive statistics of RT and surgery refusal were reported as frequency (%) relative to the total sample. Adjusted odds ratios (aOR) were calculated, and 95% confidence intervals (95%CI) for treatment refusal using logistic regression, adjusted for ethnogeographic region, age, sex, cancer staging, income, rurality, education, insurance status, comorbidity index, distance to hospital, treatment faculty type, facility U.S. region, and year of diagnosis. Population heterogeneity for treatment refusal by AA ethnogeographic region and NHPI race was assessed with likelihood ratio tests (p-heterogeneity). To control the non-random RT and surgery treatment refusal among patients and minimize treatment selection bias caused by differences in clinical characteristics between the groups, propensity score-matching (PSM) analyses were performed. Patients were propensity score-matched 1:10 (ratio of treatment refusal compared to treatment received) using the nearest neighbor-matching method. All multivariable models started with the same 14 covariates. Covariates were selected for multivariable regression models based on whether the respective model assumptions were met. Covariates were excluded for multicollinearity in regression models. Covariates were included in the PSM if they were significantly associated with radiation or surgery refusal. The matching effect of PSM was evaluated graphically with love plots and was used to visualize the distributions of different covariates after PSM balancing [14]. The threshold was set at 0.1, meaning a standardized mean difference in the covariate <0.1 would be considered satisfactory.

Overall survival was defined by the time in months between the date of diagnosis and the date of death or last follow-up. Cox proportional hazard (CPH) models were used to calculate adjusted Hazard Ratios (aHR) for mortality assessed the PSM groups based on age, sex, race, cancer stage, cancer type, income, rurality, education, insurance status, comorbidity index, distance to the hospital, facility type, facility U.S. location, and year of diagnosis.

Predictors of RT and surgery refusal were assessed through logistic regression models with calculated aOR and 95%CI. All analyses were assessed for multicollinearity using variance inflation factor tests. A *p*-value threshold of 0.05 was used to indicate statistical significance. Statistical analyses were conducted using R software, version 4.0.3, in RStudio, version 1.3.1093 (Boston, MA, USA).

## 3. Results

### 3.1. Patient Characteristics

**Figure 2:**
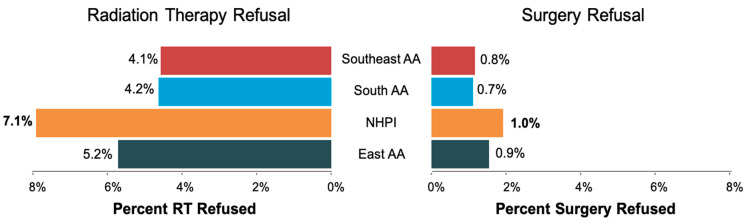
The proportions of RT and surgery refusal rates among Southeast AA, South AA, NHPI, and East AA patients. Abbreviations: NHPI = Native Hawaiian and other Pacific Islander; RT = Radiation Therapy; AA = Asian American.

The study flowchart is shown in Figure 1. The patient characteristics of 147,685 patients who met the inclusion criteria (60,117 recommended for RT and 127,276 recommended for surgery) are shown in Table 1. The median age of the RT and surgery cohort was 62 and 60 years, respectively. The median follow-up for the entire cohort was 58 months. Of 60,117 patients who were recommended RT, 2888 (4.8%) patients refused RT. Of 129,276 who were recommended surgery, 1073 (0.8%) refused surgery. RT Refusal rates by race included 5.2% of East AA, 7.1% NHPI, 4.2% of South AA, and 4.1% of Southeast AA patients. Surgery refusal rates by race were 0.9% for East AA, 1.0% for NHPI, 0.7% for South AA, and 0.8% for Southeast AA. (Figure 2). NHPI populations had the highest rate of treatment refusal for both RT and surgery refusal. Additional sociodemographic and cancer characteristics are included in Supplementary Tables S1 and S2.

### 3.2. Overall Mortality Due to Treatment Refusal

CPH models with aHR assessed PSM groups (Table 2). After matching, 2617 patients were selected for RT refusal, and 976 patients were selected for surgery refusal. On the PSM cohort, RT refusal significantly predicted higher mortality (aHR = 1.17, 95%CI = 1.08–1.27), whereas surgery refusal did not (aHR = 1.07, 95%CI = 0.95–1.20).

### 3.3. Risk and Predictors of RT and Surgery Refusal

RT and surgery refusal significantly differed between AA ethnogeographic regions and NHPI race (p-heterogeneity <0.05 for both outcomes) (Table 3). Compared to East AA, NHPI patients with cancer had a significantly higher risk of RT refusal (aOR = 1.38, 95%CI = 1.21–1.57), whereas South AA (aOR = 0.86, 95%CI = 0.76–0.97) and Southeast AA (aOR = 0.82, 95%CI = 0.74–0.91) patients had a significantly lower risk. The p-heterogeneity for RT refusal was <0.0001. Compared to East AA, NHPI patients with cancer also had a significantly higher risk of surgery refusal (aOR = 1.28, 95%CI = 1.00–1.61), whereas South AA (aOR = 0.79, 95%CI = 0.65–0.97) had a significantly lower risk and Southeast AA (aOR = 0.96, 95%CI = 0.83–1.13) had a non-significant lower risk. The p-heterogeneity for surgery refusal was 0.0093. Population heterogeneity for treatment refusal by race was assessed with likelihood ratio tests (p-heterogeneity).

When specifically evaluating the East Asian population for RT refusal, Japanese (aOR = 0.95, 95%CI = 0.82–1.10) and Korean (aOR = 0.86, 95% = 0.72–1.01) patients did not have a significantly different likelihood of refusing recommended RT treatment, compared to Chinese patients.

Predictors of RT refusal (Table S2) for all AA ethnogeographic regions and NHPI race were older patient age (aOR = 1.04, 95%CI = 1.04–1.05), female (aOR = 1.37, 95%CI = 1.15–1.64), oral cavity (aOR = 2.58, 95%CI = 1.87–3.49), and pancreas cancer (aOR = 1.62, 95%CI = 1.17–2.21) when compared to lung cancer, Midwest U.S. region (aOR = 1.22, 95%CI = 1.03–1.43), high comorbidity index (aOR = 1.92, 95%CI = 1.40–2.59), and a diagnosis between 2011 and 2017 (aOR = 1.75, 95%CI = 1.60–1.90). Oral cavity cancer was a predictor for RT refusal for NHPI (aOR = 2.52, 95%CI = 1.84–3.41), East AA (aOR = 2.52, 95%CI = 1.84–3.41), and Southeast AA (aOR = 3.17, 95%CI = 1.58–5.96) populations, whereas breast cancer was a predictor for South AA (aOR = 2.45, 95%CI = 1.30–4.95) compared to patients with lung cancer. For NHPI patients, urban–rural residence (aOR = 1.30, 95%CI = 1.02–1.63) and West US region (aOR = 1.14, 95%CI = 1.02–1.28) were unique predictors of RT refusal.

Predictors of surgery refusal (Table S3) for all AA ethnogeographic regions and NHPI race were older patient age (aOR = 1.05, 95%CI = 1.04–1.06), less education (aOR = 1.19, 95%CI = 1.04–1.35), being uninsured (aOR = 1.67, 95%CI = 1.13–2.39), high comorbidity index (aOR = 1.96, 95%CI = 1.29–2.86), and a diagnosis between 2011 and 2017 (aOR = 11.45, 95%CI = 1.27–1.66). Prostate cancer was an additional predictor for South AA (aOR = 2.18, 95%CI = 1.10–4.74) and East AA (aOR = 1.56, 95%CI = 1.14–2.14). Early-stage cancer (aOR = 1.48, 95%CI = 1.05–2.13) was an additional predictor for Southeast AA.

## 4. Discussion

The decision to refuse oncologic treatment entails a complex decision-making process that can have a significant impact on a patient’s overall mortality [2,4,5]. A previous study found that AA and NHPI race, when aggregated together, was associated with high refusal rates for RT and surgery used to treat solid malignancies [4]. However, regardless of federal guidelines to disaggregate AA and NHPI populations since 1997 [15], existing research continues to aggregate the groups together, masking disparities that exist among specific AA and NHPI populations. In this large national cohort study, we found that 4.8% of AA and NHPI patients refused RT, while 0.8% of patients refused surgery despite a cancer provider’s recommendation for treatment. Among the four AA and NHPI populations, we found that NHPI had the highest rate of both RT refusal (7.1%) and surgery refusal (1.0%), which were both significantly higher compared to East AA. We also found that RT refusal significantly predicted poorer overall survival, whereas surgery refusal did not result in a PSM cohort. Predictors for both RT and surgery treatment refusal were older patient age, higher comorbidity index, and a more recent cancer diagnosis between 2011 and 2017.

Our study found that AA and NHPI patients are specifically refusing RT more than surgery. This decision-making process and aversion to RT treatment in Asian American, Native Hawaiian, and Pacific Islander populations cannot be fully understood without acknowledging the historical and generational trauma that exists as a result of various monumental U.S. actions [1,16,17]. In our study, Native Hawaiian and Other Pacific Islander patients had the highest risk of treatment refusal for both RT and surgery. Although the specific reasons for treatment refusal were not recorded in the data used in this study, there are possible systemic and structural barriers that may interfere with accepting potentially life-saving treatment. 

Of significance, the Pacific Islands were widely used by world powers, including China, the USA, and USSR/Russia, as nuclear testing sites, leading to the release of radioactive materials into the ocean. For example, during the Cold War, 67 hydrogen bombs, equivalent to 7200 Hiroshima bombs, were detonated in the Marshall Islands leaving significant amounts of radioactive particles and radionuclides in the air, water and land [16]. The radiation fallout has been shown to bioaccumulate in the organisms via food chain cycles that have been vital sources of food and recreation for Pacific Island communities [18]. The lived experiences and multigenerational trauma that persists among these patients and communities likely influence their perceptions of medical radiation, cancer risk, trust in healthcare providers, and approach to standard and experimental cancer treatments that relate specifically to radiation [19,20].

Another reason why NHPI patients may refuse RT is due to limited access to care, predominantly for those living in Hawaiʻi and the U.S. territories of Guam and the Commonwealth of the Northern Marianas Islands (CNMI) [21,22,23]. For example, the only major facility that can provide crucial primary health services for the many islands within CNMI is located on the island of Saipan. Moreover, individuals from both the CNMI and Guam often travel to the Western U.S. for treatments and/or therapies for cancer or other chronic medical conditions that might not be available locally while also grappling with local physician shortages [24]. Even in the State of Hawaiʻi, there is only one American College of Radiology accredited radiation oncology center for the entire state, only located on one of the eight islands of Hawaiʻi [1]. For the Pacific region, only three functional radiotherapy facilities are available due to the cost of facilities, equipment maintenance costs, expertise, and the need for diagnostic, surgical, and engineering support [24]. The cost of air travel alone is upwards of U.S. $2000 for one individual, with other expenses needing to be factored in, such as accommodations and food, placing a significant financial burden on those seeking treatment [24,25]. Moreover, RT may consist of treatments 5 days per week and can continue for over a month [26], whereas surgery consists of a single-day treatment with a median postoperative hospital stay of 14 days [27]. These treatment schedules, especially traveling to and from treatment centers, can place an extra burden on patients and their family members who often accompany them to treatment [19]. This leads to one specific and impactful type of cancer toxicity, time toxicity (the time impact of cancer treatments) [28]. These may be significant contributing factors for treatment and RT-specific refusal.

Another factor to consider for RT refusal may be health literacy. In Hawaiʻi, low health literacy is more common among the rural communities, older adults, and those who identify as Filipino, Pacific Islander, or Native Hawaiian when compared to other racial/ethnic groups as measured by practical challenges often related to basic skills, such as being unable to read well enough to follow medication directions or to fill out medical forms [29,30]. Limited health literacy creates a challenge for patients when trying to understand the management of their chronic disease and the treatment options available to them, such as RT or surgery for cancer. Due to the abstract conceptualization of RT compared to surgery and the acquisition of information about RT coming from non-physician sources (i.e., family/friends, internet, etc.), the fears and perceptions surrounding RT treatment and associated treatment toxicities are common [31]. Individuals should be able to access information that is both culturally appropriate and easily understandable so they can better comprehend their medical care and make educated decisions that will impact their prognosis. This cannot be achieved by continuing to use the arbitrary grouping of AA and NHPI in healthcare and in research since each population within this umbrella term has specific cultural factors that will impact their understanding and interpretation of medical healthcare information.

Ideally, connecting patients with physicians who have similar backgrounds would help to provide culturally appropriate information, making it easier to understand their treatment. However, many populations are not adequately represented in our current physician workforce, most specifically those from NHPI populations [32]. For example, a recent study shows that NHPI representation in various subspecialties, including radiation oncology and surgery, has shown significantly declining representation over the last two decades [32]. A 2014 meta-analysis showed that increasing diversity in the healthcare workforce, specifically referencing race, ethnicity, cultural competence, and language diversity of providers, led to improvements in patient compliance and satisfaction scores while decreasing uncertainty regarding diagnosis and treatment options [33]. A potential solution is increasing early mentorship, recruitment, and retention in medicine and in various subspecialties among underrepresented populations [32,34]. This can serve as the foundation to begin providing culturally appropriate care, especially in those with such medically complicated treatments as RT or surgery, and decreasing treatment refusal rates.

For the AA population, there are also underlying historical context that must be considered in the context of RT treatment refusal. An example is the atomic bombing of Hiroshima and Nagasaki in 1945, killing over 100,000 people in their immediate deployment and tens of thousands more later dying of radiation exposure [16,35]. Due to these events, Japanese people and the public Japanese Ministry of Health, Labour and Welfare have developed “radiophobia” (fear of ionizing radiation), and consequently, have reportedly adopted an avoidant style of policy with strict and detailed regulations on radiation and radioactive materials [36]. By incorporating this apprehension into public policy, it is thus incorporated into society and culture. Moreover, in the modern era, following the well-known 2011 Fukushima power plant disaster, there was a significant increase in fear and anxiety about radiation among the Japanese population even 10 years after the event, suggesting the longevity of such negative perceptions of radiation among this population [37,38]. 

Upon conducting subset analyses disaggregating the East AA population, the Japanese did not have significantly different refusal rates compared to the other East AA (Chinese and Korean) even though these negative perceptions of radiation have been reported [36]. One potential explanation is the fact that these Japanese individuals are in the U.S. and, thus, may not conceptualize nuclear radiation trauma with medical radiation on the population level. 

Given the complexity and variability among AA and NHPI populations due to different immigration histories, colonization history, and cultural backgrounds, there are variations in healthcare accessibility, perception of health status, and attitudes toward treatments, especially related to cancer care. A recent study on racial and ethnic survival disparities in the oral cavity and laryngeal cancer found that the study population the authors identified as Asian had the best five-year overall survival (OS) when compared to Non-Hispanic White, Black, and Hispanic patients [35,39]. However, further disaggregation of the AA population into the ethnogeographic regions of East Asian, Southeast Asian, South Asian, and Pacific Islander showed significant variability in survival when stratified by race/ethnogeographic region and neighborhood socioeconomic status. The study specifically highlighted that Pacific Islander patients with oral cavity and laryngeal cancers had significantly worse five-year OS. This was an association not seen in the other AA ethnogeographic regions. Moreover, similar findings have been reported beyond head and neck cancers across the most common cancers in the U.S. [40]. For this reason, it is essential to disaggregate AA and NHPI population health data in research to fully understand the cancer health disparities of these groups and unmask important sociocultural and historical characteristics related to health outcomes [35,39].

While the strengths of this study come from the large national sample size, appropriately disaggregated NHPI data, and use of propensity-score-matched analyses, there are limitations to consider. Our study is limited by its retrospective nature with limited ability to control for confounding variables. The RT and surgery refusal variables were coded as any individual (patient, parent, or family member) refusing treatment, and therefore, the individual driving treatment refusal is unable to be determined. Furthermore, the precise reasons for treatment refusal are not definitively documented, which would otherwise add context to the numbers presented in this report. Understanding the individualized decision-making process for refusal could be integral in addressing the disparities observed with this dataset. Additionally, while the NCDB captures approximately 70% of all patients newly diagnosed with cancer, there are inherent limitations with the comprehensiveness of a large national database, particularly regarding whether areas captured by the database are enriched in the populations of interest [41]. Future studies should seek to understand individualized decision-making processes, especially among the NHPI population, ideally using a community-based participatory research approach to further discern reasons for treatment refusal and evaluate possible interventions to mitigate any associated poor survival outcomes.

## 5. Conclusions

Among AA and NHPI patients with cancer in the U.S., RT refusal was higher than surgery refusal and portended poorer OS. NHPI had the highest risk of RT refusal when compared to AA. Given that AA and NHPI are not monolithic groups, data disaggregation is necessary to understand racial and ethnic disparities for treatment refusal. Sociocultural and historical contexts of AA and NHPI populations on treatment refusal are necessary to improve cancer outcomes among these populations.

## Figures and Tables

**Table 1:** Patient Sociodemographic and cancer characteristics by recommended treatment. Abbreviations: IQR = Interquartile range; U.S. = United States; Comp Community = Comprehensive Community Cancer Center.

Characteristic	Radiation Recommended n = 60,117	Surgery Recommended n = 129,276
Follow-Up, Months, median (IQR)	62 (35–99)	60 (34–96)
Deceased	9888 (16%)	19,062 (15%)
Sex		
Male	14,623 (24%)	34,320 (27%)
Female	45,494 (76%)	94,956 (73%)
Stage		
Late	11,895 (20%)	20,000 (15%)
Early	48,222 (80%)	109,276 (85%)
Age, Years, median (IQR)	59 (49–69)	59 (49–69)
Cancer Type		
Lung	4261 (7.1%)	7157 (5.5%)
Breast	35,318 (59%)	60,702 (47%)
Colorectal	3547 (5.9%)	18,417 (14%)
Endometrial	2404 (4.0%)	8380 (6.5%)
Kidney/Bladder	285 (0.5%)	10,205 (7.9%)
Melanoma	27 (<0.1%)	948 (0.7%)
Oral Cavity	606 (1.0%)	1688 (1.3%)
Pancreas	721 (1.2%)	1578 (1.2%)
Prostate	7625 (13%)	9368 (7.2%)
Thyroid	5323 (8.9%)	10,833 (8.4%)
Income		
Higher Income	9368 (16%)	21,471 (17%)
Lower Income	46,181 (77%)	97,898 (76%)
Missing	4568 (7.6%)	9907 (7.7%)
Rurality		
Metropolitan	57,279 (95%)	122,763 (95%)
Urban–Rural	1527 (2.5%)	3432 (2.7%)
Missing	1311 (2.2%)	3081 (2.4%)
Education		
More Education	34,246 (57%)	72,395 (56%)
Less Education	21,313 (35%)	46,990 (36%)
Missing	4558 (7.6%)	9891 (7.7%)
Insurance Status		
Private Insurance	34,147 (57%)	71,454 (55%)
Medicaid/Medicare	23,477 (39%)	52,195 (40%)
Uninsured	1710 (2.8%)	3911 (3.0%)
Missing	783 (1.3%)	1716 (1.3%)
Distance To Hospital, median (IQR)	7 (3, 12)	7 (4, 12)
Missing	4550	9903
Comorbidity Index		
<2	59,652 (99%)	127,971 (99%)
3+	465 (0.8%)	1305 (1.0%)
Facility Type		
Academic	23,700 (39%)	53,656 (42%)
Community	6346 (11%)	12,864 (10.0%)
Comp. Community	19,796 (33%)	40,895 (32%)
Integrated	5376 (8.9%)	11,855 (9.2%)
Missing	4899 (8.1%)	10,006 (7.7%)
U.S. Region		
Northeast	11,429 (19%)	26,553 (21%)
Midwest	5495 (9.1%)	11,864 (9.2%)
South	8140 (14%)	18,042 (14%)
West	30,154 (50%)	62,811 (49%)
Missing	4899 (8.1%)	10,006 (7.7%)
Year of Diagnosis		
2004–2010	25,700 (43%)	52,391 (41%)
2011–2017	34,417 (57%)	76,885 (59%)

**Table 2:** Multivariable CPH analyses on a matched cohort of overall survival for aggregated AA and NHPI patients who refused RT and surgery compared to patients who were treated with RT or surgery. Vertical gray dashed line represents the reference group of treatment administered/performed. RT refusal was adjusted for patient race, age, sex, cancer type, medical comorbidities, facility type, facility location, and year of diagnosis. Surgery refusal was adjusted for the same variables except for the year of diagnosis and with the addition of cancer stage and insurance status. Abbreviations: aHR = Adjusted Hazard Ratio; 95%CI = 95% Confidence Interval; RT = Radiation Therapy.

Treatment	aHR	95% CI	Forest Plot			*p*-Value
RT Refusal			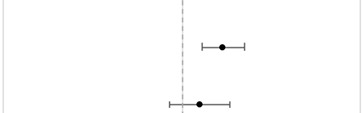			<0.001
RT Administered	1.00	Reference				
RT Refused	1.17	(1.08–1.27)				
Surgery Refusal						0.262
Surgery Performed	1.00	Reference				
Surgery Refused	1.07	(0.95–1.20)				
			0.5	1	2	
				aHR		

**Table 3:** Multivariable logistic regression of patients who refused RT and surgery by AA ethnogeographic regions and NHPI race compared to East AA. Vertical gray dashed line represents the reference group of East AA. Models were adjusted for patient race, age, sex, cancer stage, cancer type, income based on zip code, distance to hospital, education based on zip code, insurance status, medical comorbidities, facility type, facility location, and year of diagnosis. Abbreviations: aOR = Adjusted Odds Ratio; 95%CI = 95% Confidence Interval; NHPI = Native Hawaiian and Other Pacific Islander; RT = Radiation Therapy.

Characteristic	n (%)	aOR	95% CI	Forest Plot		
RT Refusal				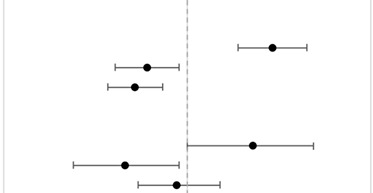		
East AA	1288 (5.2%)	—	—			
NHPI	373 (7.1%)	1.38	(1.21–1.57)			
South AA	473 (4.2%)	0.86	(0.76–0.97)			
Southeast AA	754 (4.1%)	0.82	(0.74–0.91)			
Surgery Refusal						
East AA	498 (0.9%)	—	—			
NHPI	107 (1.0%)	1.28	(1.00–1.61)			
South AA	159 (0.7%)	0.79	(0.65–0.97)			
Southeast AA	309 (0.8%)	0.96	(0.83–1.13)			
				0.5	1	2
					aOR	

## Data Availability

Data from the National Cancer Database is available in a publicly accessible repository.

## Supplementary Materials

The following supporting information can be downloaded at: https://www.mdpi.com/article/10.3390/cancers15133358/s1, Table S1. Patient demographics for RT refusal by AA ethnogeographic region and NHPI race; Table S2. Patient demographics for surgery refusal by AA ethnogeographic region and NHPI race; Table S3. Predictors of RT refusal by AA ethnogeographic region and NHPI race; Table S4. Predictors of surgery refusal by AA ethnogeographic region and NHPI race.
