# Supplementary material for: Racial Disparities among Asian American, Native Hawaiian, and Other Pacific Islander Patients with Cancer Who Refuse Recommended Radiation Therapy or Surgery
Source: Cancers (Basel). 2023 Jun 26;15(13):3358. doi: 10.3390/cancers15133358 (PMC10340289; doi:10.3390/cancers15133358)
Supplement: Supplementary file 1 [file cancers-15-03358-s001.zip › cancers-2362879-supplementary.pdf]

Supplementary Materials:

Table S1. Patient demographics for RT refusal by AA ethnogeographic region and NHPI race. Abbreviations: NHPI= Native Hawaiian and other Pacific Islands. AA=Asian Americans; Comp. Community=Comprehensive Community Cancer Program.

**Characteristic**	**Overall n = 60,117**	**East Asian n = 24,926**	**NHPI n = 5,256**	**South Asian n = 11,376**	**Southeast Asian n = 18,559**	**p-value**
**Follow-Up, Months**	62 (35, 99)	64 (35, 104)	59 (33, 98)	57 (34, 90)	62 (35, 100)	<0.001
**Deceased**	9,888 (16%)	4,291 (17%)	1,167 (22%)	1,319 (12%)	3,111 (17%)	<0.001
**Sex**						0.017
Male	14,623 (24%)	6,220 (25%)	1,241 (24%)	2,690 (24%)	4,472 (24%)	
Female	45,494 (76%)	18,706 (75%)	4,015 (76%)	8,686 (76%)	14,087 (76%)	
**Stage**						<0.001
Late	11,895 (20%)	4,670 (19%)	1,124 (21%)	2,057 (18%)	4,044 (22%)	
Early	48,222 (80%)	20,256 (81%)	4,132 (79%)	9,319 (82%)	14,515 (78%)	
Age	59 (49, 69)	60 (49, 71)	59 (50, 68)	57 (46, 67)	59 (49, 68)	<0.001
**Cancer Type**						
Lung	4,261 (7.1%)	1,992 (8.0%)	430 (8.2%)	441 (3.9%)	1,398 (7.5%)	
Breast	35,318 (59%)	14,682 (59%)	3,046 (58%)	6,831 (60%)	10,759 (58%)	
Colorectal	3,547 (5.9%)	1,539 (6.2%)	276 (5.3%)	520 (4.6%)	1,212 (6.5%)	
Endometrial	2,404 (4.0%)	757 (3.0%)	271 (5.2%)	501 (4.4%)	875 (4.7%)	
Kidney/Bladder	285 (0.5%)	151 (0.6%)	28 (0.5%)	39 (0.3%)	67 (0.4%)	
Melanoma	27 (<0.1%)	12 (<0.1%)	7 (0.1%)	0 (0%)	8 (<0.1%)	
Oral Cavity	606 (1.0%)	210 (0.8%)	50 (1.0%)	238 (2.1%)	108 (0.6%)	
Pancreas	721 (1.2%)	368 (1.5%)	70 (1.3%)	124 (1.1%)	159 (0.9%)	
Prostate	7,625 (13%)	3,312 (13%)	644 (12%)	1,416 (12%)	2,253 (12%)	
Thyroid	5,323 (8.9%)	1,903 (7.6%)	434 (8.3%)	1,266 (11%)	1,720 (9.3%)	
**Income**						<0.001
Higher Income	9,368 (16%)	3,795 (15%)	811 (15%)	1,593 (14%)	3,169 (17%)	
Lower Income	46,181 (77%)	19,582 (79%)	3,910 (74%)	8,828 (78%)	13,861 (75%)	
Missing	4,568 (7.6%)	1,549 (6.2%)	535 (10%)	955 (8.4%)	1,529 (8.2%)	
**Rurality**						<0.001
Metropolitan	57,279 (95%)	23,862 (96%)	4,845 (92%)	10,762 (95%)	17,810 (96%)	
Urban-Rural	1,527 (2.5%)	534 (2.1%)	335 (6.4%)	210 (1.8%)	448 (2.4%)	
Missing	1,311 (2.2%)	530 (2.1%)	76 (1.4%)	404 (3.6%)	301 (1.6%)	
**Education**						<0.001
More Education	34,246 (57%)	15,435 (62%)	3,053 (58%)	7,171 (63%)	8,587 (46%)	
Less Education	21,313 (35%)	7,947 (32%)	1,668 (32%)	3,254 (29%)	8,444 (45%)	
Missing	4,558 (7.6%)	1,544 (6.2%)	535 (10%)	951 (8.4%)	1,528 (8.2%)	
**Insurance Status**						<0.001
Private Insurance	34,147 (57%)	13,647 (55%)	2,923 (56%)	6,779 (60%)	10,798 (58%)	
Medicaid/Medicare	23,477 (39%)	10,456 (42%)	2,172 (41%)	3,819 (34%)	7,030 (38%)	
Uninsured	1,710 (2.8%)	523 (2.1%)	104 (2.0%)	535 (4.7%)	548 (3.0%)	
Missing	783 (1.3%)	300 (1.2%)	57 (1.1%)	243 (2.1%)	183 (1.0%)	
**Distance To Hospital**	7 (3-12)	6 (3-11)	9 (4-16)	7 (4-13)	7 (4-12)	<0.001
Unknown	4,550	1,544	533	950	1,523	
**Comorbidity Index**						<0.001
<2	59,652 (99%)	24,778 (99%)	5,171 (98%)	11,297 (99%)	18,406 (99%)	
3+	465 (0.8%)	148 (0.6%)	85 (1.6%)	79 (0.7%)	153 (0.8%)	
**Facility Type**						<0.001
Academic	23,700 (39%)	10,824 (43%)	2,144 (41%)	4,560 (40%)	6,172 (33%)	
Community	6,346 (11%)	2,538 (10%)	674 (13%)	866 (7.6%)	2,268 (12%)	
Comp. Community	19,796 (33%)	7,904 (32%)	1,562 (30%)	3,418 (30%)	6,912 (37%)	
Integrated	5,376 (8.9%)	2,063 (8.3%)	453 (8.6%)	1,034 (9.1%)	1,826 (9.8%)	
Missing	4,899 (8.1%)	1,597 (6.4%)	423 (8.0%)	1,498 (13%)	1,381 (7.4%)	
**U.S. Region**						<0.001

Northeast	11,429 (19%)	5,136 (21%)	349 (6.6%)	3,644 (32%)	2,300 (12%)	
Midwest	5,495 (9.1%)	1,519 (6.1%)	334 (6.4%)	1,944 (17%)	1,698 (9.1%)	
South	8,140 (14%)	2,480 (9.9%)	555 (11%)	2,617 (23%)	2,488 (13%)	
West	30,154 (50%)	14,194 (57%)	3,595 (68%)	1,673 (15%)	10,692 (58%)	
Missing	4,899 (8.1%)	1,597 (6.4%)	423 (8.0%)	1,498 (13%)	1,381 (7.4%)	
**Year of Diagnosis**						<0.001
2004-2010	25,700 (43%)	11,337 (45%)	2,258 (43%)	4,064 (36%)	8,041 (43%)	
2011-2017	34,417 (57%)	13,589 (55%)	2,998 (57%)	7,312 (64%)	10,518 (57%)	

Table S2. Patient demographics for surgery refusal by AA ethnogeographic region and NHPI race. Abbreviations: NHPI= Native Hawaiian and other Pacific Islands. AA=Asian Americans; Comp. Community=Comprehensive Community Cancer Program.

Characteristic	Overall n = 129,276	East Asian n = 55,029	NHPI n = 10,645	South Asian n = 24,456	Southeast Asian n = 39,146	p-value
**Follow-Up, Months**	60 (34, 96)	61 (34, 98)	58 (33, 94)	56 (32, 89)	61 (35, 96)	<0.001
**Deceased**	19,062 (15%)	8,876 (16%)	1,843 (17%)	2,605 (11%)	5,738 (15%)	<0.001
**Sex**						<0.001
Male	34,320 (27%)	15,104 (27%)	2,720 (26%)	7,153 (29%)	9,343 (24%)	
Female	94,956 (73%)	39,925 (73%)	7,925 (74%)	17,303 (71%)	29,803 (76%)	
**Stage**						<0.001
Late	20,000 (15%)	8,320 (15%)	1,699 (16%)	3,468 (14%)	6,513 (17%)	
Early	109,276 (85%)	46,709 (85%)	8,946 (84%)	20,988 (86%)	32,633 (83%)	
**Age**	59 (49, 69)	61 (50, 72)	59 (49, 68)	57 (46, 66)	59 (50, 68)	<0.001
**Cancer Type**						<0.001
Lung	7,157 (5.5%)	3,759 (6.8%)	461 (4.3%)	788 (3.2%)	2,149 (5.5%)	
Breast	60,702 (47%)	25,273 (46%)	4,805 (45%)	11,519 (47%)	19,105 (49%)	
Colorectal	18,417 (14%)	9,142 (17%)	1,119 (11%)	2,544 (10%)	5,612 (14%)	
Endometrial	8,380 (6.5%)	2,774 (5.0%)	1,190 (11%)	1,516 (6.2%)	2,900 (7.4%)	
Kidney/Bladder	10,205 (7.9%)	4,760 (8.6%)	908 (8.5%)	2,102 (8.6%)	2,435 (6.2%)	
Melanoma	948 (0.7%)	390 (0.7%)	193 (1.8%)	147 (0.6%)	218 (0.6%)	
Oral Cavity	1,688 (1.3%)	642 (1.2%)	110 (1.0%)	680 (2.8%)	256 (0.7%)	
Pancreas	1,578 (1.2%)	751 (1.4%)	144 (1.4%)	282 (1.2%)	401 (1.0%)	
Prostate	9,368 (7.2%)	3,589 (6.5%)	878 (8.2%)	2,221 (9.1%)	2,680 (6.8%)	
Thyroid	10,833 (8.4%)	3,949 (7.2%)	837 (7.9%)	2,657 (11%)	3,390 (8.7%)	
**Income**						<0.001
Higher Income	21,471 (17%)	9,300 (17%)	1,803 (17%)	3,552 (15%)	6,816 (17%)	
Lower Income	97,898 (76%)	42,432 (77%)	7,698 (72%)	18,934 (77%)	28,834 (74%)	
Missing	9,907 (7.7%)	3,297 (6.0%)	1,144 (11%)	1,970 (8.1%)	3,496 (8.9%)	
**Rurality**						<0.001
Metropolitan	122,763 (95%)	52,579 (96%)	9,591 (90%)	23,068 (94%)	37,525 (96%)	
Urban-Rural	3,432 (2.7%)	1,173 (2.1%)	838 (7.9%)	466 (1.9%)	955 (2.4%)	
Missing	3,081 (2.4%)	1,277 (2.3%)	216 (2.0%)	922 (3.8%)	666 (1.7%)	
**Education**						<0.001
More Education	72,395 (56%)	32,979 (60%)	6,054 (57%)	15,557 (64%)	17,805 (45%)	
Less Education	46,990 (36%)	18,762 (34%)	3,447 (32%)	6,934 (28%)	17,847 (46%)	
Missing	9,891 (7.7%)	3,288 (6.0%)	1,144 (11%)	1,965 (8.0%)	3,494 (8.9%)	
**Insurance Status**						<0.001
Private Insurance	71,454 (55%)	28,479 (52%)	5,820 (55%)	14,623 (60%)	22,532 (58%)	
Medicaid/Medicare	52,195 (40%)	24,646 (45%)	4,453 (42%)	8,049 (33%)	15,047 (38%)	
Uninsured	3,911 (3.0%)	1,319 (2.4%)	241 (2.3%)	1,218 (5.0%)	1,133 (2.9%)	
Missing	1,716 (1.3%)	585 (1.1%)	131 (1.2%)	566 (2.3%)	434 (1.1%)	
**Distance To Hospital**	7 (4-12)	6 (3-11)	9 (4-19)	8 (4-15)	7 (4-12)	<0.001
Unknown	9,903	3,307	1,140	1,959	3,497	
**Comorbidity Index**						<0.001
<2	127,971 (99%)	54,608 (99%)	10,401 (98%)	24,227 (99%)	38,735 (99%)	

3+	1,305 (1.0%)	421 (0.8%)	244 (2.3%)	229 (0.9%)	411 (1.0%)	
**Facility Type**						<0.001
Academic	53,656 (42%)	24,930 (45%)	4,223 (40%)	10,518 (43%)	13,985 (36%)	
Community	12,864 (10.0%)	5,463 (9.9%)	1,330 (12%)	1,784 (7.3%)	4,287 (11%)	
Comp. Community	40,895 (32%)	16,578 (30%)	3,138 (29%)	6,894 (28%)	14,285 (36%)	
Integrated	11,855 (9.2%)	4,826 (8.8%)	1,037 (9.7%)	2,223 (9.1%)	3,769 (9.6%)	
Missing	10,006 (7.7%)	3,232 (5.9%)	917 (8.6%)	3,037 (12%)	2,820 (7.2%)	
**U.S. Region**						<0.001
Northeast	26,553 (21%)	13,029 (24%)	790 (7.4%)	7,777 (32%)	4,957 (13%)	
Midwest	11,864 (9.2%)	3,308 (6.0%)	724 (6.8%)	4,275 (17%)	3,557 (9.1%)	
South	18,042 (14%)	5,436 (9.9%)	1,489 (14%)	5,884 (24%)	5,233 (13%)	
West	62,811 (49%)	30,024 (55%)	6,725 (63%)	3,483 (14%)	22,579 (58%)	
Missing	10,006 (7.7%)	3,232 (5.9%)	917 (8.6%)	3,037 (12%)	2,820 (7.2%)	
**Year of Diagnosis**						<0.001
2004-2010	52,391 (41%)	23,445 (43%)	4,155 (39%)	8,718 (36%)	16,073 (41%)	
2011-2017	76,885 (59%)	31,584 (57%)	6,490 (61%)	15,738 (64%)	23,073 (59%)	

Table S3. Predictors of RT refusal by AA ethnogeographic region and NHPI race. Abbreviations: NHPI= Native Hawaiian and other Pacific Islands. AA=Asian Americans; Comp. Community=Comprehensive Community Cancer Program.

Characteristic	NHPI			South AA			East AA			Southeast AA			Overall		
	aOR	95% CI	P	aOR	95% CI	P	aOR	95% CI	P	aOR	95% CI	P	aOR	95% CI	P
**Stage**															
Late	—	—		—	—		—	—		—	—		—	—	
Early	1.02	0.91, 1.14	0.731	1.10	0.83, 1.46	0.524	1.02	0.91, 1.14	0.731	1.05	0.85, 1.30	0.660	1.02	0.91, 1.14	0.770
**Age**	1.04	1.04, 1.05	<0.001	1.05	1.04, 1.07	<0.001	1.04	1.04, 1.05	<0.001	1.04	1.03, 1.05	<0.001	1.04	1.04, 1.05	<0.001
**Sex**															
Male	—	—		—	—		—	—		—	—		—	—	
Female	1.38	1.16, 1.66	<0.001	1.02	0.62, 1.67	0.944	1.38	1.16, 1.66	<0.001	1.52	1.06, 2.18	0.022	1.37	1.15, 1.64	<0.001
**Cancer Type**															
Lung	—	—		—	—		—	—		—	—		—	—	
Breast	1.28	1.06, 1.55	0.010	2.45	1.30, 4.95	0.008	1.28	1.06, 1.55	0.010	1.12	0.78, 1.63	0.548	1.30	1.08, 1.57	0.006
Colorectal	0.80	0.63, 1.01	0.064	1.89	0.94, 3.92	0.078	0.80	0.63, 1.01	0.064	0.76	0.48, 1.18	0.234	0.81	0.63, 1.02	0.075
Endometrial	1.11	0.86, 1.43	0.430	2.28	1.09, 4.96	0.032	1.11	0.86, 1.43	0.430	0.95	0.59, 1.54	0.850	1.13	0.88, 1.46	0.334
Kidney/Bladder	1.24	0.76, 1.91	0.363	1.97	0.43, 6.60	0.316	1.24	0.76, 1.91	0.363	1.36	0.46, 3.28	0.534	1.22	0.75, 1.89	0.399
Melanoma	1.86	0.29, 6.50	0.407				1.86	0.29, 6.50	0.407	3.38	0.18, 20.3	0.265	1.76	0.28, 6.13	0.451
Oral Cavity	2.52	1.84, 3.41	<0.001	1.81	0.76, 4.17	0.166	2.52	1.84, 3.41	<0.001	3.17	1.58, 5.96	<0.001	2.58	1.87, 3.49	<0.001
Pancreas	1.61	1.16, 2.20	0.004	1.16	0.32, 3.36	0.802	1.61	1.16, 2.20	0.004	1.55	0.74, 2.96	0.212	1.62	1.17, 2.21	0.003
Prostate	0.75	0.61, 0.94	0.010	1.07	0.57, 2.12	0.846	0.75	0.61, 0.94	0.010	0.70	0.46, 1.06	0.091	0.76	0.61, 0.95	0.014
Thyroid	0.42	0.31, 0.56	<0.001	1.00	0.46, 2.22	0.998	0.42	0.31, 0.56	<0.001	0.30	0.16, 0.52	<0.001	0.43	0.32, 0.57	<0.001
**Income**															
Higher Income	—	—					—	—					—	—	

Characteristic	NHPI			South AA			East AA			Southeast AA			Overall		
	aOR	95% CI	P	aOR	95% CI	P	aOR	95% CI	P	aOR	95% CI	P	aOR	95% CI	P
Lower Income	0.92	0.82, 1.03	0.161				0.92	0.82, 1.03	0.161				0.94	0.84, 1.05	0.270
**Rurality**															
Metropolitan	—	—		—	—		—	—		—	—		—	—	
Urban-Rural	1.30	1.02, 1.63	0.029	1.14	0.47, 2.30	0.745	1.30	1.02, 1.63	0.029	1.99	1.32, 2.89	<0.001	1.24	0.97, 1.55	0.076
**Education**															
More Education	—	—		—	—		—	—		—	—		—	—	
Less Education	1.00	0.92, 1.10	0.953	1.00	0.80, 1.24	0.978	1.00	0.92, 1.10	0.953	1.07	0.91, 1.25	0.403	1.04	0.95, 1.13	0.443
**Insurance Status**															
Private Insurance	—	—		—	—		—	—		—	—		—	—	
Medicaid/Medicare	1.03	0.94, 1.14	0.520	1.10	0.86, 1.40	0.446	1.03	0.94, 1.14	0.520	0.98	0.82, 1.18	0.834	1.02	0.93, 1.13	0.659
Uninsured	1.23	0.96, 1.57	0.092	1.06	0.64, 1.67	0.801	1.23	0.96, 1.57	0.092	1.09	0.65, 1.73	0.722	1.26	0.98, 1.60	0.067
**Comorbidity Index**															
<2	—	—		—	—		—	—		—	—		—	—	
3+	1.95	1.42, 2.63	<0.001	1.30	0.45, 3.02	0.581	1.95	1.42, 2.63	<0.001	2.18	1.19, 3.73	0.007	1.92	1.40, 2.59	<0.001
**Distance to Hospital**	1.00	1.00, 1.00	0.551	1.00	1.00, 1.00	0.743	1.00	1.00, 1.00	0.551	1.00	1.00, 1.00	0.907	1.00	1.00, 1.00	0.415
**Facility Type**															
Academic	—	—		—	—		—	—		—	—		—	—	
Community	0.85	0.74, 0.97	0.021	0.64	0.40, 0.97	0.042	0.85	0.74, 0.97	0.021	0.78	0.60, 1.00	0.050	0.86	0.75, 0.99	0.034
Comp. Community	0.95	0.86, 1.04	0.252	0.82	0.64, 1.05	0.114	0.95	0.86, 1.04	0.252	0.76	0.63, 0.91	0.003	0.97	0.88, 1.06	0.510
Integrated	0.87	0.74, 1.02	0.087	0.93	0.64, 1.34	0.723	0.87	0.74, 1.02	0.087	0.72	0.52, 0.98	0.043	0.88	0.74, 1.03	0.111
**U.S. Region**															
Northeast	—	—		—	—		—	—		—	—		—	—	
Midwest	1.20	1.02, 1.41	0.030	0.85	0.62, 1.16	0.315	1.20	1.02, 1.41	0.030	2.01	1.42, 2.85	<0.001	1.22	1.03, 1.43	0.020
South	0.93	0.79, 1.08	0.331	0.91	0.70, 1.19	0.515	0.93	0.79, 1.08	0.331	1.26	0.89, 1.79	0.201	0.93	0.80, 1.09	0.371
West	1.14	1.02, 1.28	0.020	0.83	0.60, 1.14	0.261	1.14	1.02, 1.28	0.020	1.65	1.26, 2.21	<0.001	1.09	0.97, 1.22	0.161
**Year of Diagnosis**															
2004-2010	—	—		—	—		—	—		—	—		—	—	
2011-2017	1.73	1.59, 1.89	<0.001	1.56	1.25, 1.96	<0.001	1.73	1.59, 1.89	<0.001	1.49	1.27, 1.76	<0.001	1.75	1.60, 1.90	<0.001

Table S4. Predictors of surgery refusal by AA ethnogeographic region and NHPI race. Abbreviations: NHPI= Native Hawaiian and other Pacific Islanders; AA=Asian Americans; Comp. Community=Comprehensive Community Cancer Program.

Characteristic	NHPI			South AA			East AA			Southeast AA			Overall		
	aOR	95% CI	P	aOR	95% CI	P	aOR	95% CI	P	aOR	95% CI	P	aOR	95% CI	P
**Stage**															
Late	—	—		—	—		—	—		—	—		—	—	
Early	0.72	0.44, 1.22	0.204	1.08	0.70, 1.74	0.746	1.01	0.78, 1.31	0.966	1.48	1.05, 2.13	0.031	1.09	0.91, 1.30	0.357

Characteristic	NHPI			South AA			East AA			Southeast AA			Overall		
	aOR	95% CI	P	aOR	95% CI	P	aOR	95% CI	P	aOR	95% CI	P	aOR	95% CI	P
**Age**	1.03	1.00, 1.05	0.035	1.06	1.04, 1.08	<0.001	1.05	1.04, 1.06	<0.001	1.05	1.03, 1.06	<0.001	1.05	1.04, 1.06	<0.001
**Sex**															
Male	—	—		—	—		—	—		—	—		—	—	
Female	0.68	0.34, 1.33	0.264	1.02	0.55, 1.85	0.953	0.70	0.53, 0.92	0.013	0.60	0.40, 0.89	0.011	0.70	0.57, 0.85	<0.001
**Cancer Type**															
Lung	—	—		—	—		—	—		—	—		—	—	
Breast	0.19	0.09, 0.40	<0.001	0.44	0.22, 0.98	0.033	0.42	0.31, 0.59	<0.001	0.36	0.24, 0.56	<0.001	0.37	0.30, 0.47	<0.001
Colorectal	0.21	0.09, 0.45	<0.001	0.57	0.27, 1.26	0.149	0.32	0.23, 0.43	<0.001	0.29	0.19, 0.44	<0.001	0.31	0.25, 0.39	<0.001
Endometrial	0.12	0.03, 0.35	<0.001	0.31	0.09, 0.92	0.042	0.18	0.08, 0.38	<0.001	0.17	0.07, 0.36	<0.001	0.19	0.12, 0.29	<0.001
Kidney/Bladder	0.03	0.00, 0.13	<0.001	0.21	0.07, 0.57	0.003	0.05	0.02, 0.11	<0.001	0.04	0.01, 0.11	<0.001	0.06	0.04, 0.10	<0.001
Melanoma	0.00	0.00, 1.04	0.975	0.00	0.00, 0.00	0.991	0.35	0.09, 0.96	0.080	0.41	0.07, 1.36	0.226	0.24	0.09, 0.53	0.002
Oral Cavity	0.97	0.27, 2.74	0.962	0.80	0.25, 2.25	0.691	1.13	0.62, 1.91	0.681	0.50	0.12, 1.37	0.245	0.82	0.53, 1.22	0.343
Pancreas	0.21	0.01, 1.04	0.131	0.86	0.19, 2.81	0.818	0.33	0.13, 0.71	0.010	0.20	0.03, 0.64	0.025	0.32	0.17, 0.56	<0.001
Prostate	0.62	0.31, 1.26	0.179	2.18	1.10, 4.74	0.035	1.56	1.14, 2.14	0.005	0.92	0.62, 1.37	0.676	1.23	1.00, 1.54	0.056
Thyroid	0.09	0.01, 0.35	0.002	0.00	0.00, 0.00	0.965	0.08	0.02, 0.20	<0.001	0.07	0.02, 0.18	<0.001	0.07	0.03, 0.13	<0.001
**Rurality**															
Metropolitan	—	—		—	—		—	—		—	—		—	—	
Urban-Rural	0.13	0.01, 0.60	0.042	1.50	0.35, 4.36	0.517	0.49	0.14, 1.27	0.187	1.14	0.50, 2.23	0.730	0.63	0.36, 1.03	0.083
**Education**															
More Education	—	—		—	—		—	—		—	—		—	—	
Less Education	0.82	0.51, 1.28	0.388	1.64	1.16, 2.31	0.005	1.12	0.92, 1.36	0.240	1.22	0.96, 1.57	0.105	1.19	1.04, 1.35	0.011
**Insurance Status**															
Private Insurance	—	—		—	—		—	—		—	—		—	—	
Medicaid/Medicare	1.20	0.72, 2.02	0.494	1.09	0.72, 1.66	0.677	1.05	0.84, 1.33	0.660	1.05	0.79, 1.40	0.743	1.09	0.93, 1.27	0.309
Uninsured	1.74	0.28, 6.07	0.458	1.63	0.74, 3.20	0.188	1.51	0.76, 2.71	0.198	1.89	0.91, 3.51	0.061	1.67	1.13, 2.39	0.007
**Comorbidity Index**															
<2	—	—		—	—		—	—		—	—		—	—	
3+	1.24	0.36, 3.17	0.691	0.99	0.16, 3.23	0.993	3.48	1.97, 5.72	<0.001	1.15	0.40, 2.57	0.759	1.96	1.29, 2.86	<0.001
**Distance to Hospital**	1.00	0.99, 1.00	0.424	0.98	0.97, 1.00	0.048	1.00	0.99, 1.00	0.201	1.00	1.00, 1.00	0.496	1.00	1.00, 1.00	0.062
**Facility Type**															
Academic	—	—		—	—		—	—		—	—		—	—	
Community	0.61	0.24, 1.32	0.240	1.30	0.75, 2.18	0.329	0.90	0.63, 1.26	0.548	0.88	0.56, 1.33	0.550	0.96	0.76, 1.20	0.705
Comp. Community	1.29	0.79, 2.11	0.306	0.99	0.63, 1.53	0.974	1.17	0.93, 1.46	0.172	1.09	0.82, 1.45	0.547	1.14	0.97, 1.32	0.103
Integrated	0.82	0.31, 1.85	0.665	1.28	0.67, 2.28	0.421	0.82	0.54, 1.18	0.304	1.48	0.98, 2.20	0.055	1.06	0.83, 1.34	0.626
**U.S. Region**															
Northeast	—	—		—	—		—	—		—	—		—	—	
Midwest	1.92	0.57, 7.45	0.307	0.72	0.44, 1.14	0.175	1.29	0.84, 1.92	0.227	1.21	0.73, 1.99	0.464	1.07	0.83, 1.38	0.575
South	0.89	0.26, 3.44	0.849	0.52	0.31, 0.84	0.009	1.22	0.85, 1.73	0.260	0.77	0.47, 1.27	0.311	0.81	0.64, 1.03	0.091
West	1.93	0.77, 6.45	0.213	0.57	0.33, 0.96	0.041	0.85	0.66, 1.09	0.186	1.00	0.70, 1.48	>0.999	0.87	0.74, 1.04	0.129
**Year of Diagnosis**															
2004-2010	—	—		—	—		—	—		—	—		—	—	
2011-2017	1.85	1.16, 3.02	0.011	1.31	0.92, 1.88	0.141	1.52	1.26, 1.85	<0.001	1.32	1.03, 1.70	0.027	1.45	1.27, 1.66	<0.001
